# Selecting outcome measures to validate prognostic biomarkers of paediatric mild traumatic brain injury: challenges and priorities

**DOI:** 10.3389/fneur.2025.1620178

**Published:** 2025-07-17

**Authors:** Jonathan E. Attwood, Izabelle Lövgren, Rob Forsyth, Célia Demarchi, Tony Thayanandan, Lara Prisco, Mario Ganau, Rebecca Roberts, Kate Scarff, Julia L. Newton, Gabriele C. DeLuca, Tim Lawrence

**Affiliations:** ^1^Nuffield Department of Clinical Neurosciences, University of Oxford, Oxford, United Kingdom; ^2^Oxford University Hospitals NHS Foundation Trust, Oxford, United Kingdom; ^3^The Podium Institute for Sports Medicine and Technology, Department of Engineering Science, University of Oxford, Oxford, United Kingdom; ^4^Translational and Clinical Research Institute, Newcastle University, Newcastle upon Tyne, United Kingdom; ^5^Department of Brain Sciences, Imperial College London, London, United Kingdom; ^6^Children’s Neurosciences, Evelina London Children’s Hospital, Guy’s and St Thomas’ NHS Foundation Trust, London, United Kingdom; ^7^Department of Psychiatry, University of Oxford, Oxford, United Kingdom

**Keywords:** paediatric mild traumatic brain injury, prognostic biomarkers, validation, outcomes assessment, neurodevelopment

## Abstract

Outcomes following paediatric mild traumatic brain injury (mTBI) are extremely heterogenous. While emerging biomarkers promise enhanced prognostic accuracy, a critical question remains unanswered—which outcome measures provide the most accurate assessment of injury impact? In this article, we highlight barriers to selecting appropriate outcome measures, including variability in how outcomes are defined and the wide range of assessment tools used. With reference to the most recent literature, we summarise current evidence of adverse outcomes following paediatric mTBI and highlight emerging candidate biomarkers of these outcomes. We emphasise the unique challenges associated with interpreting outcome measures in younger patients, from the impact of developmental stage and assessment timing to the influence of injury-independent factors. We assert the need to consider these obstacles when designing and interpreting mTBI biomarker studies. To realise the potential of prognostic biomarkers, future research should prioritise establishing consensus definitions, compiling a set of accessible and comprehensive outcome measures, and capturing injury-independent factors through longitudinal study designs.

## 1 Introduction

Traumatic brain injury (TBI) encompasses a wide range of conditions whereby the brain is structurally or functionally altered by a mechanical insult (1). Although TBIs are a leading cause of death and disability among young people worldwide, up to 90% are classified as ‘mild’ (i.e., Glasgow Coma Scale 13–15) (2, 3). Mild TBI (mTBI) with no structural neuroimaging abnormalities is also known as concussion (4). While mTBIs are unlikely to result in death or disability, poor medium-and long-term outcomes still occur. Indeed, a significant proportion of patients with mTBI endure persistent symptoms, ranging from headache to impaired attention and mood disturbance, all of which can be debilitating and life-changing (5).

For more than 50 years, the Glasgow Coma Scale (GCS) has been central to the assessment of TBI severity, but the variability of outcomes following mTBI highlights the need for more granular classification of these injuries (6). In a statement to this unmet need, the National Institute of Neurological Disorders and Stroke recently recommended combining clinical assessment with biomarkers, imaging, and outcome modifying factors (CBI-M) in the evaluation of TBI (7). The proposed CBI-M framework promises to transform how TBI is characterised, managed, and studied (8). However, a key challenge remains: how can we utilise these evaluations in the acute phase post-injury to distinguish who is more likely to experience adverse outcomes?

Inherent to this challenge is the need for scientific accuracy. Specifically, the distinction between biomarkers and outcome measures must be recognised. Biomarkers are measurable characteristics that indicate biological processes (9), while outcome measures capture functional impacts experienced by patients (10). Short-term outcomes, such as symptom scores, may be reliable predictors of longer-term outcomes, but this does not make them biomarkers unless they relate directly to biological processes. Recognising this distinction highlights an opportunity for both biomarkers and outcome measures collected longitudinally to be integrated into dynamic prediction models.

**Figure 1:**
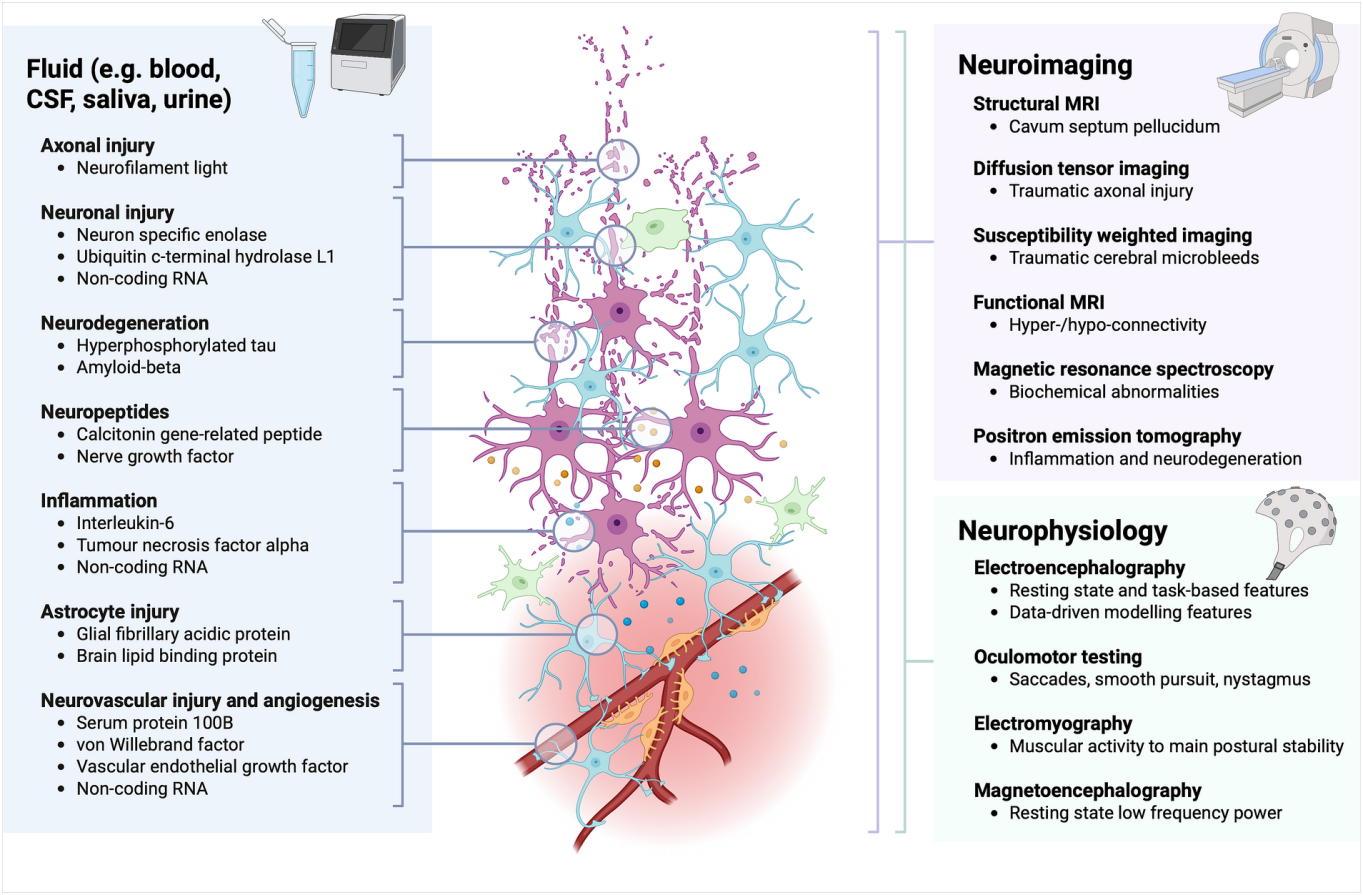
Emerging fluid, neuroimaging, and neurophysiological biomarkers of TBI. Fluid biomarkers are organised according to components of TBI pathophysiology. Neuroimaging and neurophysiological biomarkers are grouped by technique or modality.

To date, considerable research has been dedicated to identifying diagnostic and prognostic biomarkers of TBI (Figure 1) (11, 12). In contrast, relatively little attention has been given to determining *which* outcome measures these emerging biomarkers should be validated against, and what additional factors need to be accounted for. The latter is particularly important in paediatric TBI, where age at injury (13), time since injury (14), and neurodevelopmental stage (15) can all strongly influence observed outcomes. There is also substantial heterogeneity in the outcome assessment tools used, and little consensus on which measures are most appropriate, with tools developed for moderate–severe TBI (GCS 3–12) often lacking sensitivity to impairments experienced after mTBI.

To accurately validate prognostic biomarkers of mTBI, it is critical that we first understand which outcome measures provide the best assessment of the effects of an injury. In this article, we draw on the expertise of a multidisciplinary team to (i) discuss barriers to selecting outcome measures for the validation of prognostic biomarkers; (ii) summarise current evidence of adverse outcomes following paediatric mTBI and highlight emerging candidate biomarkers of these outcomes; and (iii) highlight the unique challenges in assessing and interpreting outcomes in a paediatric population. We conclude by offering recommendations for future research.

## 2 Barriers to selecting outcome measures for validating prognostic mTBI biomarkers

### 2.1 Variability in definitions across the literature

The terminology used to describe and classify brain injuries continues to pose fundamental challenges. In 2023, a consensus definition and diagnostic criteria for mTBI were published by the American Congress of Rehabilitation Medicine (ACRM), while separately the definition of sport-related concussion (SRC) was updated (4, 16). The definition of SRC still lacks diagnostic criteria, and both the meaning and relevance of the term ‘sport-related’ remains unclear. Moreover, the terms ‘mTBI’ and ‘concussion’ continue to be conflated in the literature, and the corresponding criteria used to recruit participants into studies are not always clear, limiting comparability and generalisability. Reaching consensus on an operational definition that encompasses both terms is vital to make progress in the field.

Considering outcomes, a clear definition of what constitutes abnormally prolonged recovery following mTBI is also essential to enable comparisons between studies and meta-analyses. Currently, temporal thresholds for abnormally prolonged recovery are inconsistent across the literature, spanning from 3 weeks to 3 months (17). Additionally, the domains by which recovery is assessed vary between studies, ranging from self-reported symptom resolution or resumption of usual activities to return to baseline assessment scores, among others. Given that approximately one in eight children remain symptomatic 3 months after mTBI (5), a consensus definition of abnormally prolonged recovery needs to be established. Terms used to describe this condition, including post-concussion syndrome (PCS) and persistent post-concussion symptoms (PPCS) must also be harmonised (17–19).

While diagnostic criteria for prolonged recovery have not been validated in children, a working paediatric definition has been proposed (17), nevertheless, the optimal temporal cut-off for prolonged recovery remains a key area of uncertainty, stressing the need for a better understanding of the natural evolution of mTBI in young people (20). Overcoming barriers to the long-term follow-up of paediatric patients will be central to sustaining the longitudinal studies needed to gain these insights.

### 2.2 Heterogeneity of assessment tools

**Figure 2:**
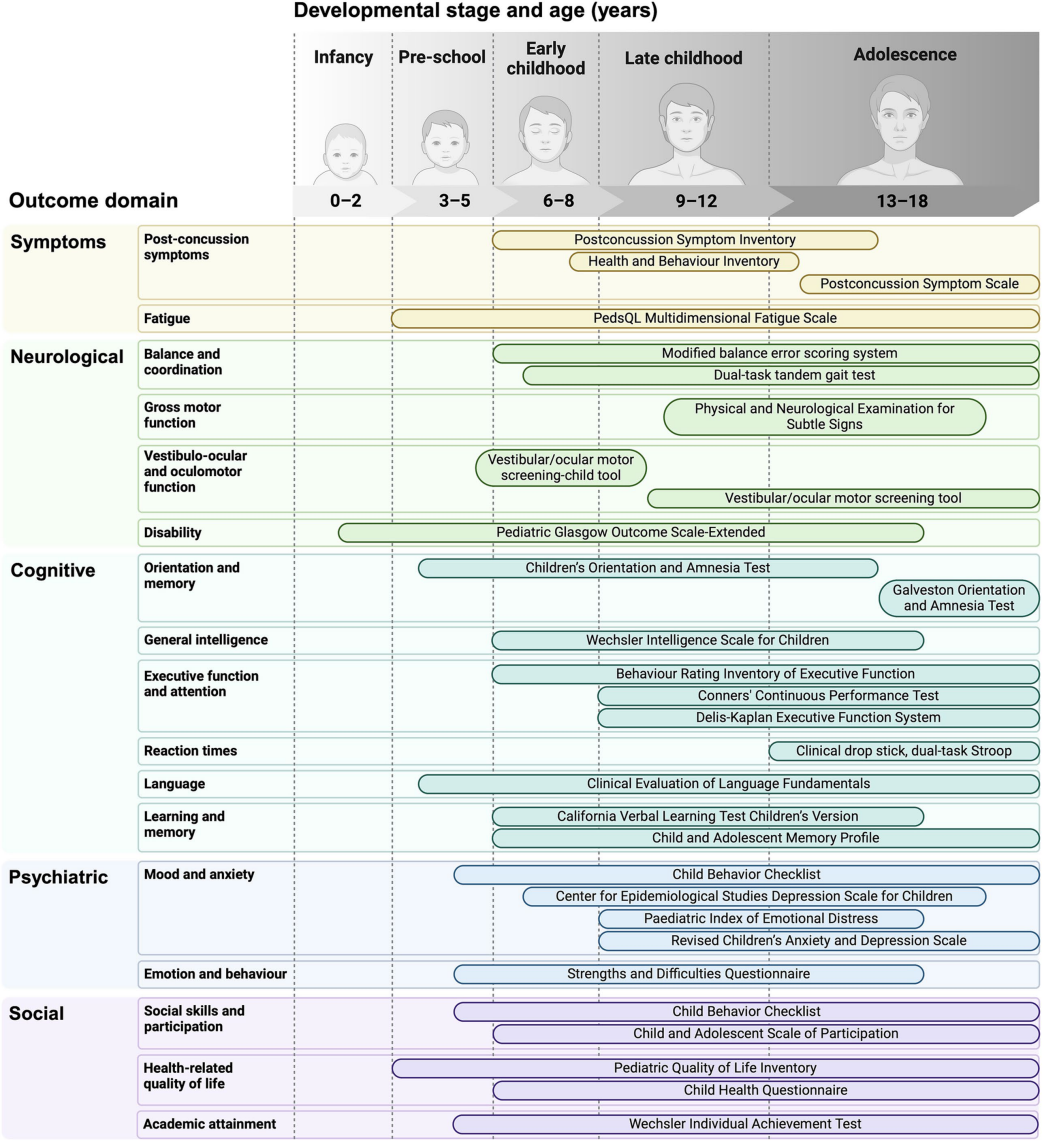
A selection of tools used throughout childhood to assess the spectrum of domains affected by mild TBI.

mTBI can produce a broad range of symptoms, and as such, those in whom symptoms persist may present to various specialties, including neurology, neuropsychology, psychiatry, and sport and exercise medicine (21). Each specialty prioritises different outcomes according to their perspective and skillset, and different specialties often employ distinct methods to measure similar outcomes (Figure 2) (22).

Even within a specialty, different sites use different tools to assess the same domains, leading to considerable heterogeneity in both clinical practice and research. Assessment tools that were originally designed for clinical use, such as the immediate post-concussion assessment and cognitive test (ImPACT), are increasingly used as outcome measures in research (23). While these tools can help to measure symptom resolution and return to baseline cognitive performance, there is limited evidence to support their use beyond the acute phase (24).

Pragmatically, the choice of tool is often guided by practical constraints, which limit the applicability of lengthy, costly, or highly technical assessments. A consensus needs to be reached about *which* assessment tools should be used to provide a comprehensive view of meaningful outcomes, while minimising time and resource burden to ensure they can be feasibly implemented in both clinical and research settings.

## 3 Current evidence of adverse outcomes following paediatric mTBI and emerging candidate biomarkers

Having highlighted the barriers posed by variable injury and outcome definitions and the heterogeneity of outcome assessment tools, in this section we summarise the range of adverse outcomes that have been demonstrated following paediatric mTBI to highlight opportunities for the development of prognostic biomarkers.

### 3.1 Neurological outcomes

Post-traumatic headache (PTH), which often resembles migraine or tension-type headache, is a common feature of prolonged recovery from mTBI, affecting over half of children with persistent symptoms (25, 26). While the pathophysiology of PTH is not fully understood, neuropeptides implicated in migraine (e.g., calcitonin gene-related peptide and nerve growth factor) have emerged as candidate biomarkers of headache after mTBI (27, 28). As no paediatric definition of PTH exists, studies validating prognostic biomarkers for PTH should use the most recent International Classification of Headache Disorders definition as their primary outcome (29). This defines PTH as headache reported to have developed within 7 days of mTBI, and persistent PTH as headache developing with 7 days and persisting for more than 3 months.

Other frequent neurological sequelae include vestibulo-ocular and oculo-motor dysfunction, resulting in dizziness, vertigo, nausea, and blurred vision (30, 31). These symptoms affect approximately one in four children with concussion and two out of three children with persistent post-concussion symptoms (30). Smooth pursuit, saccade, and vestibulo-ocular reflex abnormalities may be identifiable on examination, and could be utilised as objective outcomes measures alongside self-reported symptoms (32). Additionally, gross motor deficits including postural instability, impaired balance, and gait abnormalities, may persist up to 12 months post-injury after mTBI (33, 34).

### 3.2 Cognitive outcomes

While cognitive symptoms often arise following mTBI, there is currently no evidence of a lasting effect on intellectual abilities (23, 35, 36). Other cognitive domains, including executive function, reaction time, working memory, processing speed, and attention are variably affected in children with TBI across the spectrum of injury severity (37, 38). The balance of evidence currently suggests that these impairments tend to recover after a single mTBI, with the possible exception of executive function (23, 37, 38). In adults, a history of three or more mTBIs has been linked with impaired executive function, working memory, processing speed, and attention later in life (39, 40). However, the long-term cognitive effects of multiple mTBIs in children and young adults remains poorly understood, highlighting an important area for future research.

Advanced neuroimaging may be more apt than fluid biomarkers to predict cognitive impairments arising from altered brain network performance following mTBI. For example, volumetric abnormalities in grey and white matter structures are associated with greater cognitive difficulties after moderate–severe TBI in children (41). In adults affected by TBI, fMRI reveals hypo-connectivity within the default mode network (DMN) during performance of choice-reaction cognitive tasks, with a compensatory hyper-connectivity at rest (42). Using magnetic resonance spectroscopy (MRS), it has also been shown that frontal lobe gamma-aminobutyric acid (GABA) levels are associated with impaired working memory after mTBI (43), and subcortical levels of N-acetyl aspartate (NAA) are associated with cognitive outcomes up to 1 year after injury in children (44).

### 3.3 Psychiatric outcomes

According to parental reports, up to a quarter of young people experience significant psychological distress following mTBI (45). Children with mTBI are also more likely to exhibit internalising symptoms (e.g., anxiety, depression) and externalising symptoms (e.g., inattention, hyperactivity, aggression) compared to uninjured children (46). Longitudinal data suggest that the risk of affective and behavioural disorders, psychiatric hospitalisation, and self-harm may remain elevated for years following paediatric mTBI, compared to children with orthopaedic injuries (46, 47). Systematic reviews have found that a history of multiple mTBIs and pre-existing psychiatric illness are strong predictors of adverse psychiatric outcomes, and also highlight the importance of distinguishing between mental health symptoms and psychiatric diagnoses, noting that children with mTBI may experience symptoms which not reach diagnostic thresholds (46, 48).

There is currently limited evidence to suggest that fluid biomarkers can predict psychiatric outcomes following TBI in adults. For example, in a trial of targeted interventions for chronic psychological issues following TBI, response to treatment (measured by post-traumatic stress and overall psychological health) was predicted by composite pre-intervention serum levels of glial fibrillary acidic protein (GFAP), ubiquitin c-terminal hydrolase L1 (UCH-L1), von Willebrand factor (vWF), brain lipid-binding protein (BLBP), and vascular endothelial growth factor A (VEGF-A) (49). In terms of neuroimaging, structural abnormalities in frontal white matter are associated with novel psychiatric disorders up to 2 years after mTBI in children aged 5–14 years (50). Traumatic axonal injury (TAI) detected by diffusion tensor imaging (DTI) has been associated with psychiatric disorders following severe TBI (51), but fMRI and positron emission tomography (PET) detect alterations associated with new-onset depression and post-traumatic stress disorder (PTSD) following mTBI, with many of these studies being performed in young adults (52).

### 3.4 Social outcomes

Approximately one in eight children affected by mTBI experience persistently reduced health-related quality of life beyond one-year post-injury, and a subgroup do not return to full levels of participation in their community or school (53, 54). While a single mTBI is associated with only transiently reduced academic performance (55, 56) and no long-term effect on educational attainment, employment, or material standard of living (57), the effects of multiple mTBIs on social outcomes are not well understood. It is also important to consider whether conventional measures of these outcomes, such as academic performance, are sufficiently sensitive to injury-related impairments. Given that social disruption can have a significant and long-lasting effect on an individual’s life, further research into these outcomes is needed. Identifying robust associations between biomarkers and such complex outcomes may seem to be challenging. For example, a longitudinal study of structural brain development after mTBI in children aged 10–12 years found no differences in the thickness of cortical regions involved in social behaviour compared to uninjured controls (58). However, a recent study of military TBI in adults found that unemployment was associated with elevated plasma GFAP levels 8 years after injury (59). Further research is required to determine whether this relationship exists in paediatric mTBI cohorts.

### 3.5 Neurodegenerative outcomes

Numerous large meta-analyses have confirmed that a lifetime history of TBI is associated with a greater risk of dementia, although the risk associated with mTBI specifically remains unclear (60–63). Studies investigating the association between TBI and a post-mortem diagnosis of neurodegenerative disease have produced inconsistent findings (64). The neuropathological features of chronic traumatic encephalopathy (CTE) can be identified among former professional athletes and other individuals exposed to multiple mTBIs and repetitive head impacts (RHIs) (65–68). The duration of exposure to RHIs is associated with the extent of CTE pathology (69). However, the strength of this association is difficult to interpret in such highly selected cohorts, and the prevalence of individuals without CTE pathology after significant RHI exposure remains unclear (70, 71). Nevertheless, in one study, over 40% of brain donors aged under 30 with contact sports exposure displayed CTE pathology (72, 73). The paucity of human post-mortem material from children with RHI and mTBI limits our understanding of pathological changes in younger age groups. Including younger participants in prospective lifelong studies is therefore crucial to ensure that advances in the diagnosis and prediction of neurodegenerative outcomes are applicable across all age groups who may be at risk. Levels of hyperphosphorylated tau (p-tau) in the plasma and cerebrospinal fluid (CSF) are potential fluid biomarkers of Alzheimer’s disease (AD) and CTE (74, 75). The relation of neuroimaging biomarkers to CTE is also under investigation (76, 77).

## 4 Challenges in assessing and interpreting outcomes of paediatric mTBI

### 4.1 The impact of developmental stage and assessment timing

When assessing a paediatric population, it is essential to consider which outcomes are most relevant at different developmental stages, and to use age-appropriate assessment tools, as shown in Figure 2. In children, age at injury can significantly influence outcomes (13). Brain development is neither uniform nor linear, with different regions maturing at different rates at each developmental stage (i.e., peri-natal, infancy, pre-school, early childhood, middle childhood, late childhood, adolescence) (78). Adolescence, for example, is a time of complex neurobiological maturation (e.g., synaptic pruning, myelination) that coincides with fluctuating biological (e.g., hormonal) and psychosocial (e.g., identity formation) variables (79).

Relatively little is known about how the timing of brain injury affects these processes, or how this may differ between sexes (80–82). However, epidemiological studies support the notion that cognitive abilities are most vulnerable during critical periods of development (83). For example, infancy and middle childhood appear to be when intellectual and behavioural domains are most vulnerable to disruption by TBI, while injury during early childhood or adolescence may be more likely to affect executive function (84–88). To capture the evolution of outcomes across developmental milestones, and examine deviations as they progress over time, longitudinal studies with long follow-up periods will be needed.

### 4.2 Capturing the influence of other injury-independent factors

Clinical evaluation of injury severity is currently the mainstay of predicting adverse outcomes following mTBI (89). For instance, a higher initial symptom burden is associated with longer time to recovery (defined by return to usual activities) (13, 14, 90). However, a wide range of factors beyond injury severity can influence mTBI outcomes (17, 21). These modifying factors include the circumstances surrounding the injury, such as the degree of emotional distress experienced, as well as a wide range of injury-independent factors, including: (i) patient factors (e.g., age, developmental stage, sex, ethnicity, education, coping style, premorbid conditions and medical history) (13, 91, 92), (ii) management factors (e.g., rest, graduated return to activities, social isolation, screen time) (93–95), and (iii) environmental factors (e.g., socioeconomic status, peer relationships, family function, and other social determinants of health) (36, 96, 97).

The impact of these factors appears to accumulate over time, contributing to greater variability in cognitive, psychiatric, and social outcomes in studies with longer time since injury (21, 53, 97–99). Unless sufficiently accounted for, modifying factors are likely to confound any attempt to predict adverse outcomes following mTBI. This is particularly relevant to children and young adults, who face unique developmental challenges, often have less autonomy and capacity for self-advocacy, and may be more sensitive to environmental factors such as family functioning.

New approaches to TBI evaluation, such as the CBI-M framework, emphasise the importance of considering modifying factors at the initial assessment (100). While integrating clinical findings, biomarkers, and modifying factors entails a high degree of complexity, machine learning approaches are emerging as promising solutions (101, 102). However, further work is needed to identify the most influential modifying factors, which should then be systematically accounted for in studies seeking to validate predictive mTBI biomarkers, with reference to common data elements (21, 37, 103).

## 5 Discussion

While emerging biomarkers hold great promise for enhancing our ability to predict adverse outcomes following mTBI, careful consideration must first be given to selecting the outcome measures against which these emerging biomarkers are validated. To this end, future priorities include: (i) harmonising terminology used to describe brain injury and establishing consensus definitions and diagnostic criteria for prolonged recovery from mTBI through Delphi consensus processes; (ii) compiling a standardised set of accessible, comprehensive, and complementary outcome measure assessment tools, and (iii) developing effective methods to capture injury-independent factors in longitudinal study designs. Addressing these priorities will be essential to advance care and improve outcomes for young people affected by mTBI.

## Data Availability

The original contributions presented in the study are included in the article/supplementary material, further inquiries can be directed to the corresponding authors.
